# Cell cycle control, DNA damage repair, and apoptosis-related pathways control pre-ameloblasts differentiation during tooth development

**DOI:** 10.1186/s12864-015-1783-y

**Published:** 2015-08-12

**Authors:** Chengcheng Liu, Yulong Niu, Xuedong Zhou, Xin Xu, Yi Yang, Yan Zhang, Liwei Zheng

**Affiliations:** State Key Laboratory of Oral Diseases, West China Hospital of Stomatology, Sichuan University, Chengdu, PR China; Key Laboratory of Bio-Resources and Eco-Environment of Ministry of Education, College of Life Sciences, Sichuan University, Chengdu, PR China; Department of Orofacial Sciences, University of California, San Francisco, CA 94143 USA

**Keywords:** Ameloblasts, Differentiation, Tooth development, Transcriptional profile

## Abstract

**Background:**

Ameloblast differentiation is the most critical stepwise process in amelogenesis, and it is controlled by precise molecular events. To better understand the mechanism controlling pre-ameloblasts (PABs) differentiation into secretory ameloblasts (SABs), a more precise identification of molecules and signaling networks will elucidate the mechanisms governing enamel formation and lay a foundation for enamel regeneration.

**Results:**

We analyzed transcriptional profiles of human PABs and SABs. From a total of 28,869 analyzed transcripts, we identified 923 differentially expressed genes (DEGs) with *p* < 0.05 and Fold-change > 2. Among the DEGs, 647 genes showed elevated expression in PABs compared to SABs. Notably, 38 DEGs displayed greater than eight-fold changes. Comparative analysis revealed that highly expressed genes in PABs were involved in cell cycle control, DNA damage repair and apoptosis, while highly expressed genes in SABs were related to cell adhesion and extracellular matrix. Moreover, coexpression network analysis uncovered two highly conserved sub-networks contributing to differentiation, containing transcription regulators (RUNX2, ETV1 and ETV5), solute carrier family members (SLC15A1 and SLC7A11), enamel matrix protein (MMP20), and a polymodal excitatory ion channel (TRPA1).

**Conclusions:**

By combining comparative analysis and coexpression networks, this study provides novel biomarkers and research targets for ameloblast differentiation and the potential for their application in enamel regeneration.

**Electronic supplementary material:**

The online version of this article (doi:10.1186/s12864-015-1783-y) contains supplementary material, which is available to authorized users.

## Background

Enamel is one of the hardest mineralized tissues in vertebrates [1], and it is formed through extracellular matrix deposition by secretory ameloblasts (SABs) and the subsequent mineralization of organic matrix by mature ameloblasts (MABs) [2]. SABs and MABs are terminally differentiated cells derived from pre-ameloblasts (PABs), which are undifferentiated ameloblasts that are specified during odontogenesis [3]. PABs are elongated columnar dental epithelial cells characterized by nuclei localized near the stellate reticulum side and cytoplasm filled with organelles required for enamel protein synthesis and secretion.

Amelogenesis is a progressive differentiation process characterized by enamel formation and ameloblast differentiation, and it is regulated by various molecular and morphogenetic events. The transition of PAB to SAB is considered a hallmark of amelogenesis. Signals from the underlying dental mesenchymal compartment initiate this differentiation process by inducing PABs to SABs after basement membrane degradation during the bell stage of tooth development [4]. Differentiated SABs are tall, columnar, and polarized cells, and they can synthesize and secrete various enamel-specific proteins, such as amelogenin, ameloblastin, amelotin and enamelin [5]. These extracellular matrix proteins are essential for enamel formation and mineralization [6], and perturbation in ameloblast protein synthesis in humans results in malformed enamel [7]. After depositing full-thickness enamel matrix, ameloblasts further differentiate into short MABs to regulate enamel mineralization. Following tooth eruption, MABs undergo apoptosis [8]. Because there are no readily available ameloblast-lineage cells in human adults, they cannot restore or regenerate enamel.

Ameloblast differentiation is critical for enamel formation and enamel regeneration, thus, recent studies have focused on the molecular mechanisms of ameloblast differentiation [3, 9]. Roles of growth factors, enamel matrix proteins and transcription factors have been identified; for example, bone morphogenetic protein 2 (BMP2) and transforming growth factor-β1 (TGFβ1) were shown to induce ameloblast differentiation *in vitro* [10]. Studies on cultured tooth explants have also indicated that ameloblast differentiation is regulated by antagonistic effects between bone morphogenetic protein 4 (BMP4) and activin A [11]. In addition to these non-cell autonomous molecules, some secreted proteins, such as ameloblastin (AMBN), amelogenin (AMEL), enamelin (ENAM), and enamel proteinases such as matrix metalloproteinase 20 (MMP20) play key roles in full ameloblast differentiation [8]. The stage-specific expression of these genes is controlled by specific transcription factors. For example, the overexpression of Runt-related transcription factor (Runx2) and Distal-less homeobox 3 (Dlx3) result in the up-regulation of *AMEL* and *ENAM* mRNA levels [12]. These studies identified fundamental molecular events that control ameloblast differentiation and enamel regeneration. However, the differentiation from PAB to SAB is likely controlled by a precise molecular network like most cell differentiation processes. Therefore, further study of the transcriptional profile during ameloblast differentiation is important to identify novel biomarkers and research targets for the future investigation of tooth development and regeneration.

Here, we performed a comprehensive analysis of PABs and SABs transcriptional profiles from individual gene transcriptional levels to functional categories of enriched genes. This strategy revealed that the transition from PABs to SABs coincided with diverse DEGs involved in multiple pathways. Particularly, genes encoding growth factors, signaling molecules, transcription factors and their corresponding receptors, as well as membrane transport proteins were specifically expressed, and these molecules likely govern the biological processes essential for ameloblast differentiation and amelogenesis, including cell cycle, cell proliferation, cell adhesion, apoptosis and biomineral tissue development. Finally, we used bioinformatic approaches to explore significant coexpression networks and identify novel coexpression associations of central hubs in these networks.

## Results

### PAB and SAB morphology and gene marker characterization

**Fig. 1:**
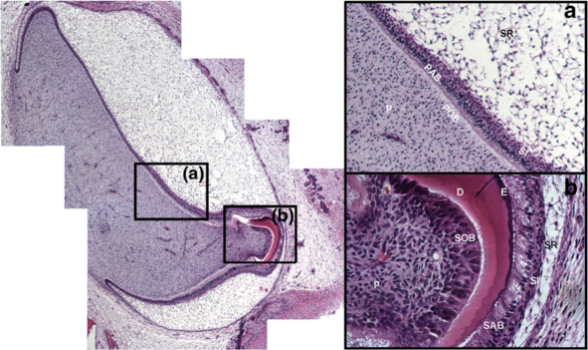
Identification of ameloblasts from a developing human incisor for laser capture microdissection. Left panel: H&E staining of a developing human incisor. Right panel: **a** Magnification of **(a)** in left panel. Pre-ameloblasts (PAB) were polarized inner enamel epithelial cells that directly contacted the basement membrane and were adjacent to polarized odontoblasts. **b** Magnification of **(b)** in left panel. Secretory ameloblasts (SAB) were identified as polarized epithelial cells in direct contact with the enamel matrix. (PAB: preameloblast, SAB: secretory ameloblast, POB: preodontoblast, SOB: secretory odontoblast, D: dentin, E: enamel, SR: Stellate reticulum, SI: Stratum intermedium, P: dental pulp)

**Fig. 2:**
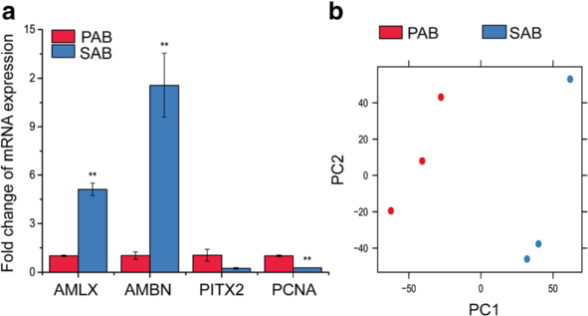
**a**. Characterization of different stages of micro-dissected human dental epithelial cells. *AMLX* and *AMBN* expression levels were up-regulated as cells differentiated, while *PCNA* expression decreased in SABs compared to PABs. **p < 0.01. *PITX2* expression was up-regulated in PABs compared to SABs. **b**. PCA clustering showed a clear divergence of PABs and SABs

To isolate PABs and SABs from human tooth buds, we performed rapid H&E staining to identify each cell type by location and morphology, then collected the cells by laser capture microdissection (LCM) (Fig. 1). In addition, we performed quantitative real-time PCR (*q*PCR) on micro-dissected cells (PABs and SABs) from human incisors to examine the expression of four genes known to be involved in ameloblast differentiation. As expected, SABs more highly expressed *AMLX* and *AMBN* compared to PABs, and PABs expressed higher levels of *PCNA* compared to SABs. *PITX2* expression was up-regulated in PABs compared to SABs (Fig. 2a).

### Distinct transcriptional patterns in PABs and SABs

To generate the transcriptional profiles of PABs and SABs, we performed Affymetrix Human Genome 1.0ST Arrays on RNA from laser-captured cells. First, to obtain the whole transcriptional changes related to the differentiation from PABs to SABs, we applied principal component analysis (PCA) to the normalized microarray data. As anticipated, when the group number was set as 2, the transcriptional landscapes of the PABs and SABs separated into independent clusters, indicating distinct gene expression patterns for the two cell types (Fig. 2b). By comparing the probe intensity of 28,869 transcripts we identified a total of 923 DEGs with a threshold fold change (FC) > 2 and *p* < 0.05 between PABs and SABs. Among these DEGs, 647 genes were upregulated in PABs compared to SABs and 276 genes were upregulated in SABs compared to PABs. (See Additional file 1 for a full list of identified genes). Furthermore, 38 genes were dramatically changed (FC > 8) between PABs and SABs (Table 1).

**Table 1:** Most significant DEGs between PABs and SABs

Gene Symbol	Description	Fold-change	*p*-value	FDR
Genes up-regulated in PAB				
TRPA1	transient receptor potential cation channel, subfamily A, member 1	20.22	1.58E-11	3.17E-07
WEE1	WEE1 homolog (S. pombe)	15.47	2.78E-03	6.62E-02
ETV1	ets variant 1	15.36	2.57E-09	1.43E-05
HIST1H2BM	histone cluster 1, H2bm	14.79	7.93E-09	3.30E-05
SPC25	SPC25, NDC80 kinetochore complex component, homolog (S. cerevisiae)	13.44	1.18E-09	9.82E-06
FAM177B	family with sequence similarity 177, member B	11.90	4.67E-08	8.64E-05
CDKN3	cyclin-dependent kinase inhibitor 3	11.83	6.02E-06	1.63E-03
FAM111B	family with sequence similarity 111, member B	11.67	5.55E-09	2.64E-05
DLGAP5	discs, large (Drosophila) homolog-associated protein 5	11.41	3.36E-07	2.95E-04
BIRC5	baculoviral IAP repeat containing 5	10.78	3.03E-06	1.08E-03
NUSAP1	nucleolar and spindle associated protein 1	10.78	1.90E-08	5.74E-05
ETV5	ets variant 5	10.48	1.42E-07	1.90E-04
TOP2A	topoisomerase (DNA) II alpha 170 kDa	10.48	6.66E-08	1.06E-04
BRIP1	BRCA1 interacting protein C-terminal helicase 1	10.41	1.78E-06	7.79E-04
PLK4	polo-like kinase 4	10.02	5.30E-05	6.03E-03
CENPF	centromere protein F, 350/400 kDa (mitosin)	9.81	6.31E-08	1.05E-04
EFEMP1	EGF containing fibulin-like extracellular matrix protein 1	9.39	8.76E-06	2.07E-03
ASPM	asp (abnormal spindle) homolog, microcephaly associated (Drosophila)	9.33	6.40E-10	7.10E-06
SNORD30	small nucleolar RNA, C/D box 30	9.13	8.45E-04	3.23E-02
FAM9A	family with sequence similarity 9, member A	9.12	1.29E-08	4.76E-05
RAD51AP1	RAD51 associated protein 1	8.96	7.47E-07	4.69E-04
CENPK	centromere protein K	8.63	3.69E-07	3.15E-04
DTL	denticleless homolog (Drosophila)	8.60	2.70E-06	1.01E-03
KIF11	kinesin family member 11	8.53	1.59E-07	1.99E-04
HELLS	helicase, lymphoid-specific	8.32	2.52E-07	2.37E-04
SEMA3E	sema domain, immunoglobulin domain (Ig), short basic domain, secreted, (semaphorin) 3E	8.18	1.96E-04	1.35E-02
Genes up-regulated in SAB				
MMP20	matrix metallopeptidase 20	-26.41	9.20E-07	4.91E-04
SLC15A1	solute carrier family 15 (oligopeptide transporter), member 1	-21.58	6.42E-06	1.72E-03
SLC7A11	solute carrier family 7 (anionic amino acid transporter light chain, xc- system), member 11	-18.49	1.24E-05	2.53E-03
RGS13	regulator of G-protein signaling 13	-15.51	7.68E-06	1.94E-03
SPHKAP	SPHK1 interactor, AKAP domain containing	-11.86	3.14E-05	4.59E-03
ENAM	enamelin	-10.78	3.16E-03	7.06E-02
HPGD	hydroxyprostaglandin dehydrogenase 15-(NAD)	-10.14	4.38E-06	1.34E-03
LAMC2	laminin, gamma 2	-10.07	2.56E-05	4.10E-03
NMNAT2	nicotinamide nucleotide adenylyltransferase 2	-10.02	1.47E-05	2.78E-03
DSPP	dentin sialophosphoprotein	-9.32	1.41E-02	1.58E-01
FAM20A	family with sequence similarity 20, member A	-9.13	8.58E-07	4.91E-04
DMP1	dentin matrix acidic phosphoprotein 1	-8.73	4.71E-04	2.29E-02

### KEGG pathway analysis of differentially expressed genes

**Fig. 3:**
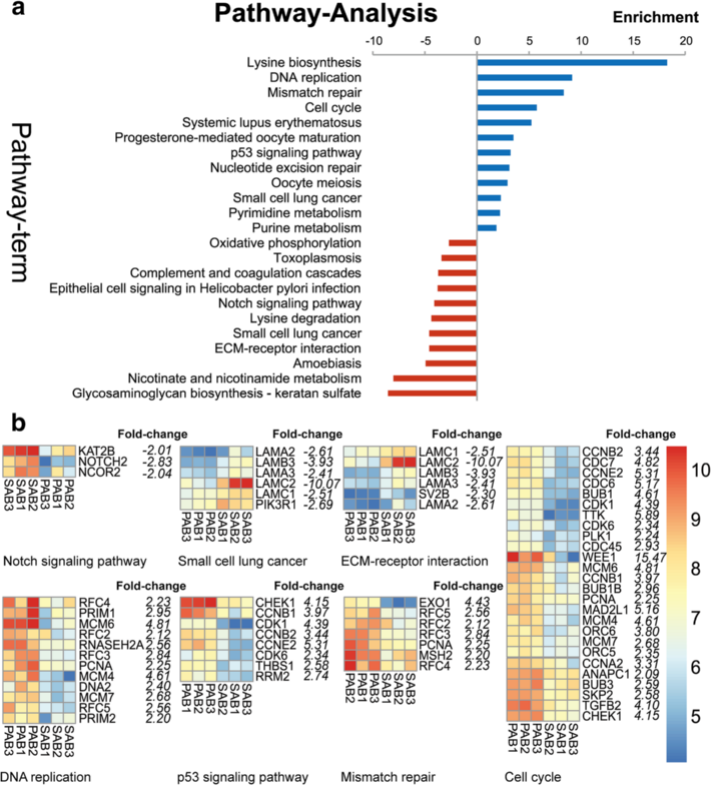
KEGG pathway analysis. **a**. KEGG pathway enrichment analysis was performed by Fisher exact test. Significantly enriched KEGG pathways (*p* < 0.05) are presented. For each KEGG pathway, the bar shows the fold-enrichment of the pathway. **b**. The expression intensity of genes in the selected KEGG pathway. The italic values on the right of gene names were the fold changes of corresponding genes in PABs compared to SABs

We annotated the DEGs identified in the comparison of PABs to SABs using the Kyoto Encyclopedia of Genes and Genomes (KEGG) pathway. We assigned DEGs to 280 pathways, and we identified a total of 23 significantly enriched pathways (threshold *p*-value < 0.05) (Fig. 3a). We applied size-dependent enrichment analysis to map each gene cluster into pathways. Cluster maps of DNA replication, mismatch repair, cell cycle, p53 signaling, Notch signaling, small cell lung cancer and ECM-receptor interaction pathways are shown in Fig. 3b.

### Up-regulated genes in PABs control cell cycle, DNA damage and apoptosis

Our microarray results revealed that the up-regulated genes in PABs compared to SABs are primarily involved in cell cycling, cell proliferation, apoptosis and DNA mismatch repair. Cyclin-dependent kinases (CDKs) have been implicated as involved in the cell cycle control system, including chromosome condensation, nuclear envelop breakdown and spindle assembly, by phosphorylating intracellular proteins [13, 14]. Genes encoding CDK family members, including *CDC2*, *CDKL2*, *CDK6*, and *CABLES1*, showed a higher level of expression in PABs compared to SABs. Since it is known that CDKs tightly bind to cyclins to form cyclin/CDK complexes, which in turn activate CDKs and trigger cell-cycle progression [15], we next investigated the corresponding cyclin family members. In accordance with the detected increase of CDKs, genes encoding cyclins (for instance, *CCNA2*, *CCNB1*, *CCNB2*, and *CCNE2*) and the cyclins interacted protein *CCNBLIP1* (cyclin B1 interacting protein) were highly expressed in PABs compared to SABs. Interestingly, several negative cyclin/CDK complex regulators, *WEE1*, cyclin-dependent kinase inhibitor 3 (*CDKN3*) and CDK inhibitor (CKI), were also up-regulated in PABs compared to SABs. These inhibitors, WEE1 for example, phosphorylate the kinase active site to inhibit cyclin/CDK complex activity (Additional file 2: Table S3) [16, 17].

Another noteworthy feature in PABs is the DNA damage, which is an important factor in cell cycle progression. DNA damage initiates a signaling pathway, and the cell cycle control system can detect signals to arrest the cell cycle at late G1 or G2/M checkpoints [18]. We found that *MDM2* expression was increased in PABs compared to SABs. MDM2 is a critical regulator of the cell cycle, as it ubiquitinates P53, targeting it for destruction by the proteasome [19]. Moreover, members of the DNA mismatch repair pathway, including *MSH2*, *PMS2l5*, *RMI1* and *EXO1,* were enriched in PABs compared to SABs (Additional file 2: Table S3) [20, 21].

Notably, *CASP6* and *CASP8AP2* expression was increased in PABs compared to SABs (Additional file 2: Table S3), and they encode the apoptosis-related executioner caspase (Caspase 6) and initiator caspase (Caspase 8), respectively [22].

Taken together, the significantly up-regulated genes in PABs will work coordinately to form a sophisticated network that tightly controls the cell cycle, DNA damage and apoptosis, altogether contributing to ensuring that PABs remain in a proper state to support differentiation.

### SABs are enriched for genes associated with extracellular matrix and adhesion

Amelogenin, ameloblastin, enamelin and amelotin are four EMPs, which are highly expressed during the secretory and early maturation stages of amelogenesis [23]. They are subsequently degraded step-wise from the matrix by MMP20 and kallikrein 4 (KLK4) during the ameloblast secretory stage and maturation stage, respectively [2]. Among them, amelogenin is the most abundant protein in enamel matrix and is continuously expressed throughout amelogenesis. Ameloblastin is the second most abundant EMP, and it localizes between enamel rods. Enamelin is the largest enamel matrix protein of developing teeth and comprises approximately 5 % of total EMPs. As expected, genes encoding AMBN, ENAM, AMTN and MMP20 were more highly expressed in SAB cells compared to PAB cells [6] (Additional file 2: Table S3). However, *AMLX* and *KLK4* expression did not significantly differ between PABs and SABs. Other genes encoding proteins involved in biomineral tissue development (*DSPP*, *DMP1*, *PHEX*, *ALPL* and *MMP16*) [24–27] were also more highly expressed in SABs compared to PABs (Additional file 2: Table S3).

In addition to genes encoding secreted proteins, SABs were enriched in five genes encoding laminins, including *LAMC1*, *LAMC2*, *LAMB3*, *LAMA3* and *LAMA2* (Additional file 2: Table S3). Laminins are primary basal lamina components, and they play key roles in differentiation, migration and adhesion [28]. Laminin alpha5 (*LAMA5*)-null mice have been shown to display aberrant cusp formation and dental epithelium proliferation [29].

Apart from adhering to the extracellular matrix, cell-cell adhesions also play an indispensable role in stabilizing the cellular and tissue structure. Cell-cell adhesion sites are strengthened by the connected underlying cytoskeleton, which then forms a network across several cells. In vertebrates, cell-cell adhesion is primarily mediated by the cadherin superfamily, which are important for cell rearrangements during tissue morphogenesis [30]. We found that cadherin superfamily members *CDH8* and *CDH13* were enriched in SABs compared to PABs (Additional file 2: Table S3). In addition, adhesion molecule with Ig-like domain 2 (*AMIGO2*) and *IGJ* expression were both elevated in SABs compared to PABs (Additional file 2: Table S3) [31, 32].

### Differential expression of transcription factors and signaling pathways between PABs and SABs

By comparing PAB and SAB transcriptional profiles, we found that several growth factors, signaling molecules and transcription factors were differentially expressed between the two cell types. The transforming growth factor family members transforming growth factor β2 (*TGFβ2*) was more highly expressed in PABs compared to SABs, while bone morphogenetic protein 8A (*BMP8A*) was more highly expressed in SABs compared to PABs. Several other growth factors were differentially expressed between the cell types, suggesting that they may be critical regulators in ameloblast differentiation. One fibroblast growth factor family member (*FGF20*) was increased in SABs, while fibroblast growth factor 9 (*FGF9*) and nuclear factor I b (*NFIB*) expression were decreased in SABs compared to PABs. Interestingly, several negative regulators of the fibroblast growth factor receptor signaling pathway (*SPRY1*, *SPRY2*, *SPRY4*, *THBS1* and *SULF1*) [33–36] were enriched in PABs compared to SABs (Additional file 2: Table S3).

Notch signaling and MAPK signaling contribute to cell differentiation during tissue development [37, 38]. In this study, we found that *Notch 2* and *MAPK6* expression was increased in SABs compared to PABs. However, two other MAPK members (*MAPK10* and *MAPK14*) were enriched in PABs compared to SABs. Moreover, the distalless related homeodomain proteins (*Dlx5*) and transcription factor AP-2 alpha (*TFAP2A*) were highly expressed in PABs compared to SABs (Additional file 2: Table S3). Two G protein-coupled receptor pathway members (*GPR110* and *GPSM2*) were more highly expressed in PABs. In addition, three interleukin genes were differentially expressed between PABs and SABs. *IL17* receptor (*IL17RD*) expression increased, while *IL1* receptor (*IL1R1*) and *IL33* expression decreased in PABs compared to SABs (Additional file 2: Table S3).

### Transporters and ion channels are differentially expressed between PABs and SABs

Transcellular Ca^2+^ transport plays an important part in the formation of calcium hydroxyapatite during amelogenesis. Previous studies have shown that stromal interaction molecule 1 (*STIM1*) and solute carrier family 24, member 4 (*SLC24A4*) were critical ion transport systems in enamel maturation [39]. We found that in addition to *SLC24A4*, nine other solute carrier family members (*SLC15A1*, *SLC7A11*, *SLC41A2*, *SLC13A5*, SLC4A4, *SLC17A5*, *SLC25A33* and *SLC45A4*) were enriched in SABs compared to PABs. In contrast, six family members (*SLC26A4*, *SLC4A7*, *SLC35A3*, *SLC27A2, SLC16A10* and *SLC35F1*) were increased in PABs compared to SABs (Additional file 2: Table S3).

Channels are another major class of membrane transport proteins [40]. We found that three genes encoding transient receptor potential (TRP) channels, including *TRPM7*, *TRPA1* and *TRPC1,* were differentially expressed between the two cell types. TRPM7 is essential for many cellular processes, including proliferation, survival, differentiation, growth, and migration [41]. Its expression increased in SABs compared to PABs, while *TRPA1* and *TRPC1* were more highly expressed in PABs. Strikingly, *TRPA1* expression was more than 16 fold higher in PABs than SABs (Additional file 2: Table S3).

Other ion transport-associated genes that were differentially expressed include members of Na-K-ATPase (*ATP1B1*) and V-ATPase (*ATP6V0D1* and *ATP6V0A1*), which were all increased in SABs compared to PABs (Additional file 2: Table S3).

### Genome-wide coexpression network construction identified ameloblast development-associated modules

Different cell types synthesize different sets of proteins, so, to a large extent, gene expression defines the cell differentiation program. However, rather than functioning as completely independent components, genes interface in intricate ways to create a network. In particular, signaling networks precisely regulate diverse cellular events, including cell cycle, morphogenesis, proliferation, cell adhesion, and programmed cell death [42].

**Fig. 4:**
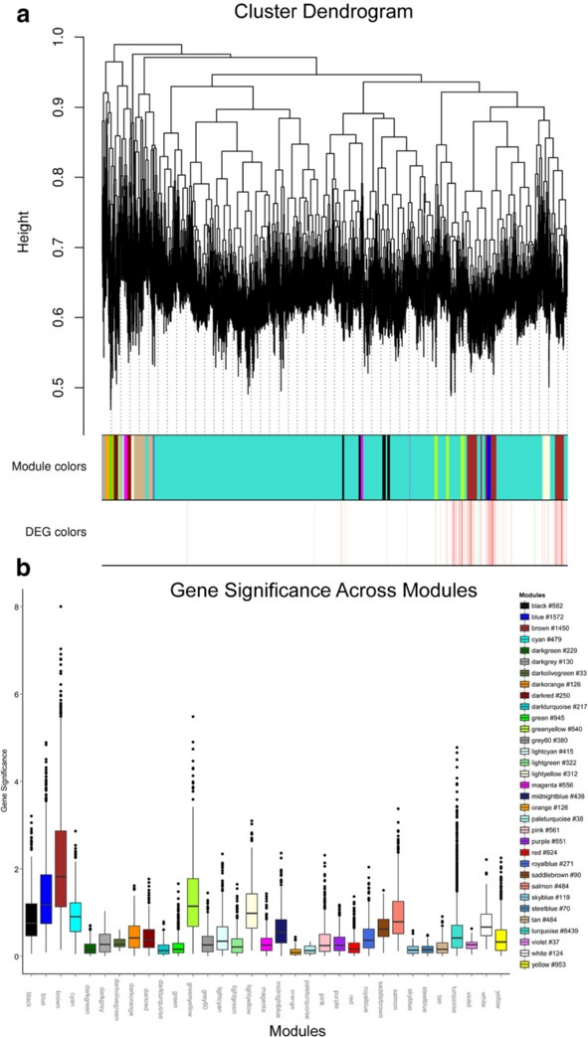
Gene coexpression modules and assigned module colors. **a**. Clustering dendrogram of genes with the x-axis corresponding to each gene and the y-axis representing the dissimilarity based on topological overlap of the whole genome throughout ameloblast differentiation. Top color bar: modules were assigned colors corresponding to different dendrogram branches detected by a cluster algorithm. Second color bar: each gene was annotated with DEG information such that significantly up-regulated genes in PABs are marked with red bands and genes down-regulated in PABs marked with green bands. **b**. Boxplot depicting gene significance defined as -log10(*p-value*) of each module. The left legend indicates module colors and the number of genes (stars with a number sign) in the corresponding module (for example, 1527 genes in the blue module)

**Fig. 5:**
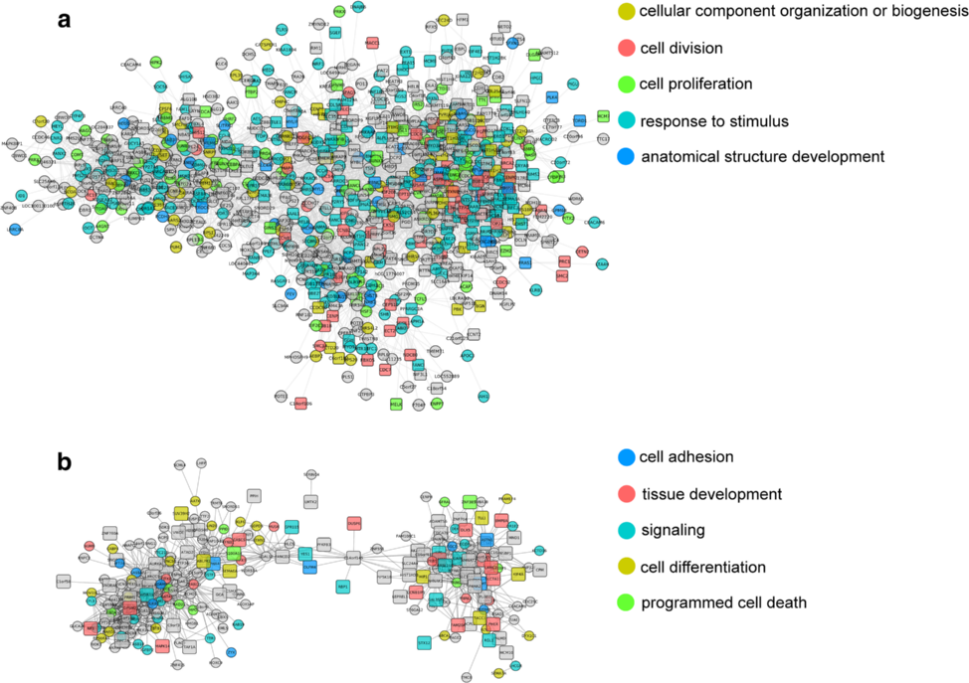
Gene coexpression networks of modules. Global network visualization of the top two modules “brown” (**a**) and “blue” (**b**) that are highly correlated with gene significance. Each node corresponds to a gene, and each edge joining two nodes indicates the connection determined by the adjacency. The nodes in boxes represent DEGs and circles indicate genes without significant differential expression between PABs and SABs. Nodes are also marked with colors to indicate their representative GO biological processes, and grey nodes are those not included in either of the GO terms. The left legend in each network shows colors and corresponding GO terms

To further explore specific networks that orchestrate ameloblast differentiation and identify the gene coexpression associations of DEGs in these networks, we used weighted gene coexpression network analysis (WGCNA) to identify modules containing highly coexpressed genes. The coexpression network was based on the correlation values between a pair of genes across microarray data. As shown in Fig. 4a, we identified a total of 33 conserved modules and assigned them with unique colors. To determine how modules correlated with the differentiation of preameloblasts to secretory ameloblasts, we defined the gene signature measure (GS) as a transformation from *p*-values for testing differential gene expression between PABs and SABs (details in Methods). In particular, brown and blue modules (named by color) were enriched with high GS values (Fig. 4b). After mapping the DEGs to the coexpression modules, we found that brown and blue modules contained a significant number of DEGs (413 in brown and 252 in blue), as shown in Fig. 4a (second color bar). These data indicate that these two modules were highly related to differential gene expression in ameloblast differentiation. We also visualized the global coexpression network in brown and blue modules with a proper adjacency threshold (Fig. 5), and the detailed interaction list is in Additional file 3: Table S4 and S5.

### Brown and blue module hub genes

**Fig. 6:**
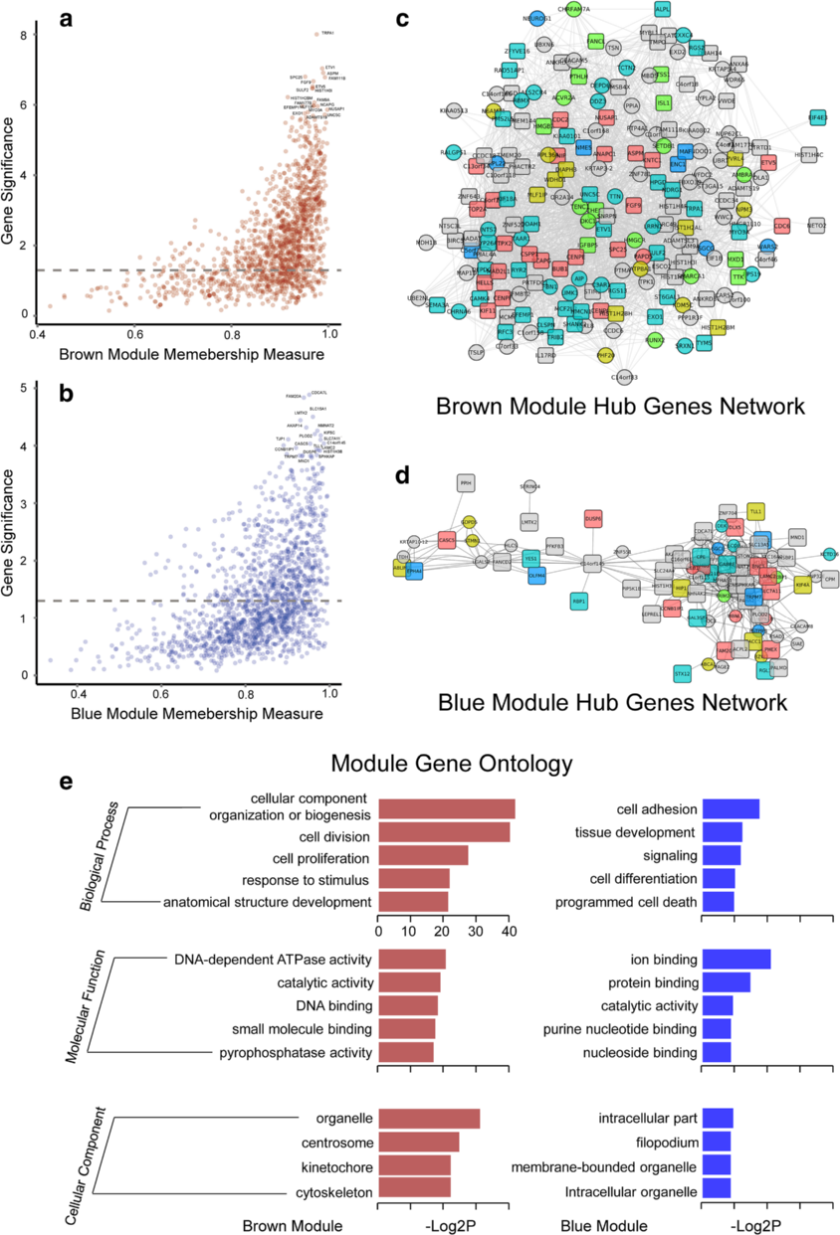
Hub gene selection and gene ontology analysis of brown and blue modules. **a** and **b** are the scatterplots between module membership measure (x-axis) and the gene significance of the “brown” and “blue” module. The top 20 genes with both the highest gene significance and membership measure larger than 0.9 were chosen as “hub genes”, and they are annotated with their gene symbols. The grey dashed line in the plot is the threshold for choosing significantly expressed genes, and the threshold value is –log10(0.05). **c** and **d** are the hub gene network visualizations for the “brown” and “blue” modules. The colors of the hub genes and their directly connected genes indicate the GO terms from Fig. 5 (a) and (b). **e**. Barplots represent enriched GO terms of all genes included in the “brown” (left with brown color) and “blue” (right with blue color) networks, and –log2(*p*-value) represents the relative enrichment of each GO term

To investigate the biological functions of the WGCNA modules, we selected the highly connected “hub genes” within each module. Due to their high degrees of connectivity, intramodular hub genes were centrally located in coexpression networks and may play an important role in their module’s cellular function and signaling pathways [43, 44]. We defined the module membership measure (MM) as the correlation between the gene expression and module eigengene. We selected the top 20 genes with high MM (>0.9) and GS values as reprehensive hub genes for the brown and blue modules (Fig. 6a and b) [45].

In the brown module, there was a significant correlation (*cor* = 0.50, *p-value* = 1.50 × 10^−92^) between the intramodular connectivity and the gene significance, indicating the potential gene expression pattern in the brown module (Fig. 6a). Interestingly, *RUNX2* was one of the highly connected genes in the brown module (MM = 0.93), which is consistent with a previous study that showed RUNX2 was a key regulator in ameloblast differentiation by regulating amelogenin and enamelin expression [12]. More importantly, we detected *RUNX2* directly connected to 18 genes, including *EXO1* (adjacency = 0.420), *SULF2* (adjacency = 0.422) and *ETV1* (adjacency = 0.423) (Fig. 6c and Additional file 3: Table S4). These genes may be *RUNX2* partners during ameloblast differentiation. In addition, we identified other genes encoding RUNX proteins (*RUNX1* and *RUNX3*) in the brown module. The hub genes *RUNX1* (MM = 0.99) and *RUNX3* (MM = 0.95) directly connected to 21 and 8 genes in the brown module, respectively (Fig. 6c and Additional file 3: Table S4). This was consistent with previous reports that they act as master developmental regulators [46] and indicated their function and partners in ameloblast differentiation. Another highly connected gene *MMP20* (MM = 0.94) was significantly increased (FC = 25) in SABs and was associated with anatomical structure development. This result was consistent with a recent study that MMP20 facilitates ameloblast movement by cleaving ameloblast cell-cell contacts [47]. *FGF9* (MM = 0.97) was also located in the center of the coexpression network (Fig. 6c), which suggests that they also play an important role in ameloblast differentiation. This was in line with their role in transcription regulation throughout development [48].

We also detected another group of hub genes including *TRPA1*, *ETV1*, *ASPM*, *SPC25* and *NUSAP1* (Additional file 3: Table S6). Although these genes have not been previously associated with ameloblast differentiation, their functions have been demonstrated in previous studies. TRPA1 is a stress sensor [49], and ETV1 is an ETS transcription factor required for retinal neuron differentiation [50]. Their appearance as hub genes in the brown module and up-regulation (FC > 8) in PABs highly suggest that they play critical functions in ameloblast differentiation. Notably, *TRPA1* and *ETV1* directly interacted with each other (adjacency = 0.428), and both of them had direct interactions with three other mitosis-associated hub genes (*ASPM*, *SPC25* and *NUSAP1*) (Fig. 6c and Additional file 3: Table S4 and S6) [51–53]. It is therefore reasonable to infer that *TRPA1* and *ETV1* influence ameloblast differentiation by regulating cell division. Strikingly, *ETV1* also directly interacted with *RUNX2*, suggesting that *ETV1* plays pleiotropic roles in ameloblast differentiation.

Moreover, *ETV5* was a hub gene with the highest MM (0.97), but it was not directly connected with *ETV1*. Rather, it was surrounded by another hub gene in the brown module (*FAM111B*) with an adjacency of 0.42 (Fig. 6c and Additional file 3: Table S4). These data suggest that the two ETS transcription factors are not regulated in the same fashion and may therefore perform distinct physiological functions. This is consistent with the recent discovery that *ETV4* and *ETV5* often perform similar functions during morphogenesis, whereas *ETV1* has distinct roles [54].

Similar to the brown module*,* we observed that the intramodular connectivity and GS were highly correlated in the blue module (*cor* = 0.49, *p* = 1.00 × 10^−90^), as shown in Fig. 6b. *SLC15A1*, *SLC7A11* and *SPHKAP* were hubs in the blue module with a module membership of 0.96, 0.98 and 0.97, respectively. We found that *SLC15A1* directly interacted with 27 genes and *SLC7A11* directly connected with 24 genes (Fig. 6d and Additional file 3: Table S5). Interestingly, *SPHKAP* showed a coexpression relationship with both *SLC15A1* (adjacency = 0.414) and *SLC7A11* (adjacency = 0.414) (Fig. 6d and Additional file 3: Table S5). *SPHKAP* is annotated as an SPHK1-interacting protein. However, its role is still unclear. The interaction between *SPHKAP* and solute carrier family members (*SLC15A1* and *SLC7A11*) suggests that it may function in solute transport during ameloblast differentiation.

*LAMC2* showed a high MM of 0.97 in the blue module, suggesting that it has an indispensable function (Additional file 3: Table S7). By performing GO analysis, we found that it was involved in tissue development (Fig. 6d), which could suggest that laminin plays an important role in ameloblast differentiation. Though the exact function of laminin in ameloblast differentiation has not been reported, its function as an extracellular matrix component suggests that it is involved in cell proliferation and differentiation during tooth morphogenesis [55].

As an epithelial-to-mesenchymal (EMT) transcription factor, zinc finger E-box binding protein 1 (ZEB1) has been implicated in neural crest cell biology [56]. Though *ZEB1* was not detected as a hub gene in the blue module, it directly connected with the *SLC15A1* hub gene with an adjacency of 0.41, and it was surrounded by DEGs in the blue module hub gene network (Fig. 6d), indicating its potential role in ameloblast differentiation.

Interestingly, we also identified two BMP family members in the two coexpression modules. However, *BMP6* (MM = 0.95) localized to the brown module (Additional file 3: Table S4 and S6), and *BMP8A* (MM = 0.95) localized to the blue module (Additional file 3: Table S5 and S7), indicating an independent regulatory function in ameloblast differentiation.

### Gene Ontology (GO) analysis reveals gene association and biological processes essential to ameloblast differentiation

To functionally characterize genes in the brown and blue modules, we performed GO analysis. Genes associated with cellular component organization or biogenesis, cell division and proliferation were significantly enriched in the brown module, whereas genes related to ion binding, cell adhesion, and tissue development were enriched in the blue module (Fig. 6e), which indicated a striking difference in the functions of the two modules. The brown module closely associated with cell division and proliferation, and almost all of the hub genes in the brown module were up-regulated in PABs compared to SABs. While the blue module was likely related to cell adhesion and tissue development, its hub genes were upregulated in SABs compared to PABs. Collectively, these results suggested that the blue and brown modules play critical and distinct roles in ameloblast differentiation. We also identified several genes with high MM but low GS in both modules (Fig. 6a and b), suggesting that they were closely related to certain functions of the corresponding modules but that they not serve as DEGs. By separately plotting the genes within each GO term (Additional file 4), we found that the genes with high MM and low GS were functionally annotated. For example, those genes in the brown module were annotated as being involved in response to stimulus, while those in the blue module were annotated as functioning in cell differentiation. We speculated that these genes may act by interacting with hub genes or as indispensable components in biological pathways, and they should be examined as potential players in ameloblast differentiation.

### *q*PCR validation of microarray results

To validate microarray results, we confirmed the transcriptional levels of 12 genes in PABs and SABs by *q*PCR, including seven DEGs and *AMLX*, *KRT14*, *RUNX2*, *KLK4* and *STAT2* (Table 2). Most of the genes exhibited differential expression in *q*PCR that was consistent with the microarray data, indicating good concordance of both methods.

**Table 2:** Results of microarray and *q*PCR experiments

Gene name	Description	Gene expression Microarray			*q*PCR	
		FC	*p*-Value	FDR	FC	*p*-Value
AMLX	amelogenin	−1.68	4.09E-02	4.36E-01	−33.27	5.22E-03
AMBN	ameloblastin	−4.80	1.44E-02	1.59E-01	−5.71	3.29E-03
ENAM	enamelin	−10.78	3.16E-03	7.06E-02	−1.48	6.13E-02
MMP20	matrix metallopeptidase 20	−26.41	9.20E-07	4.91E-04	−15.22	4.52E-03
KRT14	keratin 14	1.14	5.89E-01	8.53E-01	24.33	1.11E-02
RUNX2	runt related transcription factor 2	−1.68	1.37E-03	1.08E-01	−1.26	3.72E-01
KLK4	kallikrein-related peptidase 4	−1.18	1.75E-01	6.25E-01	1.03	7.18E-01
STAT2	signal transducer and activator of transcription 2	−1.28	2.03E-01	6.48E-01	−1.01	8.37E-01
ETV1	ets variant 1	15.36	2.57E-09	1.43E-05	25.01	3.97E-02
SLC15A1	solute carrier family 15, member 1	−21.58	6.42E-06	1.72E-03	−14.82	8.21E-04
SLC7A11	solute carrier family 7, member 11	−18.49	1.24E-05	2.53E-03	−6.19	1.32E-02
DSPP	dentin sialophosphoprotein	−9.32	1.41E-02	1.58E-01	−4.64	6.38E-03

## Discussion

Ameloblasts are a tooth-specific cell type, and they secrete extracellular matrix proteins and deposit teeth enamel [6]. Full ameloblast differentiation occurs by three distinct processes: the presecretory, secretory and maturation stages [4]. The sequential differentiation of ameloblast lineage cells requires signals from the underlying mesenchymal compartment and dentinal matrix. Ameloblasts differentiate in a niche composed of secretory proteins, enzymes, signaling molecules and other factors. Ameloblast differentiation is defined by the shift from preameloblast to secretory ameloblast, and it can significantly influence enamel matrix protein secretion and mineralization initiation. This process is regulated by a complex signaling network composed of signaling molecules, their receptors and transcriptional regulators [3]. However, the field lacks a full understanding of the transcriptional program underlying ameloblast differentiation. In this study, we aimed to identify molecular events governing the shift from PABs to SABs and gene coexpression networks that control ameloblast maturation.

Comparison of transcriptional profiles of PAB and SAB together with KEGG pathway analysis revealed that PAB cells express genes that allow them to control cell cycle, repair DNA mismatch and eliminate abnormal, misplaced, or nonfunctional cells by apoptosis, which are necessary quality-control processes for PAB differentiation. In contrast, pathways enriched in SAB were primarily related to extracellular matrix and cell adhesion. It is widely accepted that SABs secrete EMPs into the enamel matrix, which is primarily composed of three structural proteins (AMELX, AMBN and ENAM). These proteins are degraded step-wise from the matrix by MMP20 and KLK4 at ameloblast secretory stage and maturation stage, respectively [2]. In this study, we identified the increased expression of four secretory ameloblast-specific genes (*AMBN*, *AMTN*, *ENAM* and *MMP20*) in SABs by microarray, which is consistent with previous studies and further validated by our LCM and microarray approach. Although microarray failed to detect *AMELX* enrichment in SABs, we confirmed its increased expression in SABs by real time PCR. This discrepancy may be due to the detection limit of microarrays, as fold-changes observed by microarray are usually lower than those observed by real-time PCR [57]. In addition, we identified five other genes (*DSPP*, *DMP1*, *PHEX*, *ALPL* and *MMP16*) related to biomineral tissue development enriched in SABs, suggesting that these genes may regulate ameloblast maturation and enamel formation.

In addition to genes encoding extracellular matrix proteins, we also noted the enrichment of cadherin and laminin genes in SABs. Cadherins control the selective cell sorting, and the upregulation and downregulation of specific cadherins correlate with specific steps in embryonic development [58]. Previous studies have shown increased expression of E-cadherin and N-cadherin in PABs and SABs, respectively [47]. We found that cadherin superfamily genes (*CDH8*, *CDH13* and *CDHR3*) and laminin genes (*LAMC1*, *LAMC2*, *LAMB3*, *LAMA3* and *LAMA2*) were more highly expressed in SABs compared to PABs, suggesting that they may regulate secretory ameloblast cell rearrangement and morphogenesis. However, this is the first implication that they may function in amelogenesis; thus, their precise function in ameloblast differentiation require further investigation.

Some signaling pathways are critical for animal development, including receptor tyrosine kinase (RTK), transforming growth factor family, Wnt, Hedgehog and Notch. In addition, JAK/STAT, nuclear hormone receptor, and G-protein-coupled receptor pathways are important in some developmental processes [42, 59]. Terminal ameloblast differentiation is regulated by the interactions between the epithelium and mesenchyme. PAB differentiation can be influenced by odontoblasts signals, such as TGFβ1, BMP2 and BMP4 [3, 60]. Our study provides proof that members of growth factors (*BMP8A*, *TGFβ2*, *NFIB*, *FGF9* and *FGF20*), signaling pathways (*MAPK6*, *MAPK10*, *MAPK14* and *Notch2*), G protein coupled receptor pathways (*GPR110* and *GPSM2*) and transcription factors (*Dlx5* and *TFAP2A*) participate in the differentiation of PABs to SABs.

Differentially expressed genes and KEGG pathway analysis revealed key genes and metabolic characteristics that may regulate PAB differentiation to SABs. Further coexpression network analysis allowed us to identify modules of highly correlated genes from whole transcriptome profiles and identify hub genes and their expression associations during ameloblast differentiation. Moreover, a functional enrichment analysis of the brown and blue modules revealed basic functional pathways preserved in these modules.

## Conclusions

In this study, we found that the PAB to SAB differentiation relies on a highly regulated network of interactions between conserved signal transduction pathways, including members of the BMP/TGF-β, Notch, MAPK pathways to coordinate all aspects of ameloblasts in intracellular processes and their social contexts. Specifically, expression of genes associated with cell cycle control, DNA damage repair, and apoptosis pathways regulate pre-ameloblast maturation during tooth development. SAB cells are regulated by several signaling pathways that control enamel matrix protein secretion and cell adhesion, which are critical for enamel formation and cell-cell interactions. Furthermore, the application of bioinformatic analysis allowed us to explore potential key genes and gene-associations involved in ameloblast differentiation. These findings will aid in the design of new strategies to promote ameloblast differentiation in tooth regeneration and tissue engineering. However, further experiments are required to confirm the roles of these genes and their interacting genes. Although transcriptional controls are paramount for most gene expression, these genes and pathways can also be regulated by a complex array of genetic (e.g., RNA splicing and selecting), epigenetic (e.g., histone modification), non-coding RNA (e.g., microRNA and long non-coding RNA) and exogenous signaling factors that serve to guide cell fate and behavior during development and differentiation.

## Methods

### Ethics statement

All human tissues were collected from legally aborted fetuses at West China Women and Children’s Hospital under the approved guidelines of Sichuan University. The written informed consent of all human subjects was obtained. The study and the consent procedure were approved by the Ethical Committees of West China School of Stomatology, Sichuan University and State Key Laboratory of Oral Diseases.

### Sample collection

Human tooth buds (18-22 weeks) were obtained from fetal cadaver tissue within three hours after legal abortion under the guidelines of the West China School of Stomatology, Sichuan University Committee on Human Research. Teeth were dissected from the mandibles under a laminar flow hood, embedded in OCT compound (Sakura Finetek, Torrance, CA, USA), and cryosectioned at 10-μm thickness. These sections were used for LCM.

### Laser capture microdissection (LCM)

A PALM MicroBeam system (Carl Zeiss Micro Imaging, Inc. Thornwood, NY, USA) laser capture microscope was used for cell dissection [61, 62]. All reagents used for LCM and RNA purification were prepared in RNase-free water. Cryosectioned human tooth organs were mounted on polyethylene naphthalate (PEN) foil glass slides. After rapid hematoxylin-eosin (H&E) staining, slides were air-dried, and dental epithelial cells were identified by location and morphology. PABs and SABs were separately dissected and catapulted to Adhesive Cap (Carl Zeiss MicroImaging Gmbh, München, Germany) containing ß-Mercaptoethanol-supplemented RLT buffer (RNeasy Plus Micro Kit, RNeasy Lysis Buffer, Qiagen, Valencia, CA, USA). The caps containing buffer and cells were incubated upside-down at room temperature (RT) for 30 min and then briefly centrifuged to collect the buffer and cell complexes. RNA purification was performed with an RNeasy Plus Micro Kit (Qiagen), and reverse transcription was performed with SuperScript™ III First-Strand Synthesis System (Invitrogen Corporation, Carlsbad, CA, USA) following the manufacturer’s instructions.

### Microarray procedures

Affymetrix Human Genome 1.0ST Arrays (Affymetrix, Santa Clara, CA, USA) covering 28,869 transcripts in the human genome were employed for transcriptional profiling and performed by Affymetrix. Three to four pre-ameloblast and secretory ameloblast samples collected by LCM were pooled for each array. RNA samples were extracted and purified by RNeasy Mini kits (Qiagen and Rnase-Free DNase Set) as described previously [63]. A Nanodrop ND 1000 spectrophotometer (Thermo Fisher Scientific, Pittsburgh, PA, USA) was used to determine total RNA concentrations, and an Agilent 2100 Bioanalyzer (Agilent Technologies, Santa Clara, CA, USA) was used to evaluate RNA quality [64] Both RNA samples were of acceptable quality (Average RIN of 9.0 and 8.0 for PABs and SABs, respectively), and there was no significant difference between their qualities. For each replicate, 100 ng of total RNA was amplified and labeled using the Affymetrix Whole-Transcript (WT) Sense Target Labeling Protocol without rRNA reduction. Array hybridization, washing, and scanning were performed according to the protocol described in WT Sense Target Labeling Assay Manual.

### Microarray expression analysis

The R/Bioconductor Package “Affy” was used to perform gene expression quartile normalization to adjust the marginal distribution of each sample. We used the “limma” package to identify DEGs [65]. Genes with fold-change greater than 2 and *p* < 0.05 between PABs and SABs were considered statistically significant.

### KEGG pathway analysis

The complete list of human pathways was retrieved from the KEGG Pathway Database (http://www.genome.jp/kegg/). A functional gene enrichment analysis was performed based on the KEGG pathways [66]. We applied Fisher’s exact test to determine whether a set of DEGs was selectively enriched in a pathway. KEGG pathways containing at least two DEGs with *p*-value less than 0.05 were considered significant.

### Weighted gene coexpression network analysis (WGCNA)

The WGCNA package in R was used to construct unsigned coexpression networks [45, 67]. After filtering raw data without gene function annotation, 22,247 probes were used to construct the pair-wise Pearson’s correlation coefficient matrix. Next, the correlation matrix was transformed to an adjacency matrix by a soft power adjacency function provided by Bin Zhang and Steven Horvath [45]. The scale-free topology criterion method was used to choose the power parameter *β*. Specifically, *β* = 16 was used as a balance between the maximization of scale-free topology model fit (topological scale R square was 0.73) and the number of connections. The adjacency value for each pair of gene was defined as raising the absolute value of their Pearson’s correlation coefficient to the power *β*, and the adjacency values ranged from 0 to 1. By using the adjacency matrix, the topological overlap measure, which is a similarity measurement, was calculated for each pair of genes. The topological overlap-based dissimilarity was then used for clustering and dynamic tree cutting with the minimum module size of 30 and merged tree-cut height of 0.25 [68]. A group of genes that were tightly gathered into the same cluster was considered a coexpression module. The *blockwiseModules* function in WGCNA R package was used to automatically select modules in PABs and SABs, and the maximum block size was set to 10,000 due to the limitation of computer memory.

The *T*-statistics-based *p-value* yielded from “limma” R package [65] was used for each gene as a criterion to choose proper modules associated with ameloblast differentiation. The gene significance (GS) should be a positive value calculated by the formula: GS = −log10 (*p*-value). The top two modules with the highest GS values (equal to the enrichment of more DEGs) were selected and identified as the brown and blue modules. To make a representative visualization of the correlation network, only the strongest linkages were drawn (correlation was greater than 0.95). Correlation networks of the brown module and blue module were generated in Cytoscape with adjacency thresholds of 0.42 and 0.41, respectively [69].

### Hub gene selection and gene ontology analysis

The most highly connected genes, which were also well-known hub genes, were used to represent the expression profiles and biological characters of the entire module. The module membership measure (MM) for a gene was defined as the correlation between the eigengene of the module and the gene expression; therefore, the MM was also named the module eigengene based connectivity (kME) [45]. As we used the *p-value* based GS for module selection, the top 20 genes with both highest GS and MM (0.9 or greater) were defined as intramodular hub genes. Subnetworks consisting of intramodular hub genes and their directly connected genes were also plotted.

Functional enrichment analysis for each selected module was conducted by the R/Bioconductor package clusterProfiler [70]. The background gene set was the total list of genes used for coexpression network construction. The threshold level to determine the significance of all the GO enrichment was set as *p* < 0.05.

### Microarray data accession

All raw microarray data were submitted to the NCBI Gene Expression Omnibus database with the accession number GSE59214, which can now be viewed through the link http://www.ncbi.nlm.nih.gov/geo/query/acc.cgi?acc=GSE59214

### Quantitative real-time PCR

For cell-type confirmation after LCM, *AMLX*, *AMBN*, *PITX2*, and *PCNA* expression was examined by quantitative real-time PCR in an ABI 7500 system (Applied Biosystems, Foster City, CA, USA). For microarray data validation, we examined the transcriptional levels of 12 genes in PABs and SABs by *q*PCR. RNA was extracted with the same method as described for microarray. The relative abundance of mRNA transcripts was quantified relative to GAPDH levels using the ∆∆CT method [71]. Primers and probe sets including an endogenous GAPDH control were purchased from Applied Biosystems (Applied Biosystems, Foster City, CA, USA). The corresponding arithmetic formulas used are as follows: ΔCTcondition = CTtarget gene − CTendogeneous control and ΔΔCT = ΔCTcondition1 − ΔCTcondition2. For example, we compared the gene expression fold change between different cell types. Take amelogenin expression in PABs and SABs as an example, ΔCT_PAB_ = CT_amelogenin_ at PAB-CT_GAPDH_ at PAB, ΔCT_SAB_ = CT_amelogenin_ at SAB-CT_GAPDH_ at SAB, ΔΔCT = ΔCT_SAB_-ΔCT_PAB_. The fold change of SAB to PAB is calculated as Fold_SAB to PAB_ = 2^(−ΔΔCT)^ (amelogenin expression at PAB was used as a baseline value of 1). Data were compared by one-way ANOVA followed by the post hoc Tukey’s test.

## Additional files

Additional file 1:
**Differentially expressed genes between PABs and SABs.** **Table S1.** Genes significantly up-regulated in PABs. **Table S2.** Genes significantly up-regulated in SABs. (XLS 194 kb) Additional file 2: Table S3. Expression of selected genes in PABs compared to SABs. (XLS 40 kb) Additional file 3: Table S4 and Table S5. Selected connections, including gene symbols of nodes and adjacency, in the brown and blue modules. **Table S6** and **Table S7**: Selected connections with membership measure and gene significance in the brown and blue modules. (XLS 933 kb) Additional file 4:
**Scatterplots between module membership measure (x-axis) and gene significance of GO terms in the brown module (A-E) and blue module (F-J).** The grey dashed line in the plot is the threshold for choosing significantly expressed genes, and the threshold value is –log10(0.05). (TIFF 1712 kb)
