# Supplementary figures and images for: Cell cycle control, DNA damage repair, and apoptosis-related pathways control pre-ameloblasts differentiation during tooth development
Source: BMC Genomics. 2015 Aug 12;16(1):592. doi: 10.1186/s12864-015-1783-y (PMC4534026; doi:10.1186/s12864-015-1783-y)

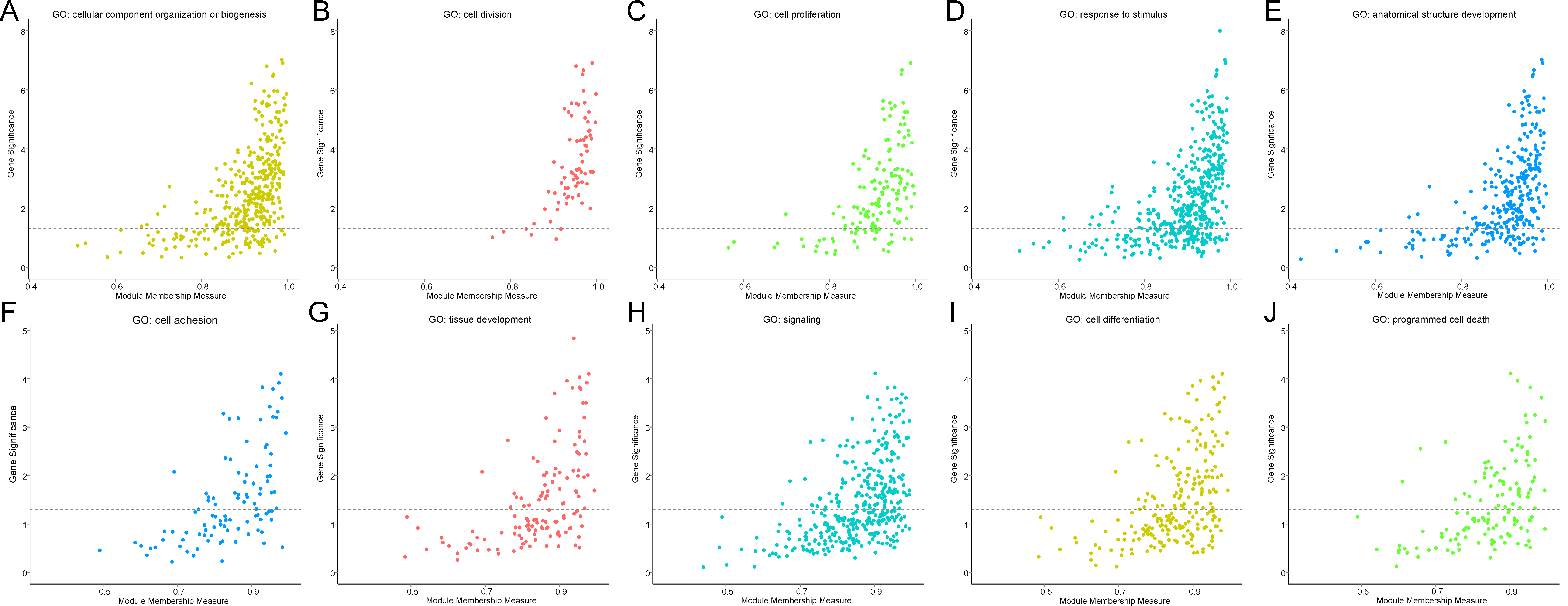

Supplement: Additional file 4: — Scatterplots between module membership measure (x-axis) and gene significance of GO terms in the brown module (A-E) and blue module (F-J). The grey dashed line in the plot is the threshold for choosing significantly expressed genes, and the threshold value is –log10(0.05). (TIFF 1712 kb) [file 12864_2015_1783_MOESM4_ESM.tiff]
